# Long-term changes of bacterial and viral compositions in the intestine of a recovered *Clostridium difficile* patient after fecal microbiota transplantation

**DOI:** 10.1101/mcs.a000448

**Published:** 2016-01

**Authors:** Felix Broecker, Jochen Klumpp, Markus Schuppler, Giancarlo Russo, Luc Biedermann, Michael Hombach, Gerhard Rogler, Karin Moelling

**Affiliations:** 1 Institute of Medical Microbiology, University of Zurich, 8006 Zurich, Switzerland;; 2 Max Planck Institute for Molecular Genetics, 14195 Berlin, Germany;; 3 Institute of Food, Nutrition and Health, ETH Zurich, 8096 Zurich, Switzerland;; 4 Functional Genomics Center Zurich, University of Zurich and ETH Zurich, 8057 Zurich, Switzerland;; 5 Division of Gastroenterology and Hepatology, University Hospital Zurich, 8006 Zurich, Switzerland;

**Keywords:** recurrent infection of the gastrointestinal tract

## Abstract

Fecal microbiota transplantation (FMT) is an effective treatment for recurrent *Clostridium difficile* infections (RCDIs). However, long-term effects on the patients’ gut microbiota and the role of viruses remain to be elucidated. Here, we characterized bacterial and viral microbiota in the feces of a cured RCDI patient at various time points until 4.5 yr post-FMT compared with the stool donor. Feces were subjected to DNA sequencing to characterize bacteria and double-stranded DNA (dsDNA) viruses including phages. The patient's microbial communities varied over time and showed little overall similarity to the donor until 7 mo post-FMT, indicating ongoing gut microbiota adaption in this time period. After 4.5 yr, the patient's bacteria attained donor-like compositions at phylum, class, and order levels with similar bacterial diversity. Differences in the bacterial communities between donor and patient after 4.5 yr were seen at lower taxonomic levels. *C. difficile* remained undetectable throughout the entire timespan. This demonstrated sustainable donor feces engraftment and verified long-term therapeutic success of FMT on the molecular level. Full engraftment apparently required longer than previously acknowledged, suggesting the implementation of year-long patient follow-up periods into clinical practice. The identified dsDNA viruses were mainly Caudovirales phages. Unexpectedly, sequences related to giant algae–infecting *Chlorella* viruses were also detected. Our findings indicate that intestinal viruses may be implicated in the establishment of gut microbiota. Therefore, virome analyses should be included in gut microbiota studies to determine the roles of phages and other viruses—such as *Chlorella* viruses—in human health and disease, particularly during RCDI.

## INTRODUCTION

*Clostridium difficile* is the leading cause of hospital-acquired infectious diarrhea (Lo Vecchio and Zacur 2012). This bacterium capitalizes on antibiotic disruption of the normal microbiota to colonize the intestine, causing disease mainly by secreted toxins (Lo Vecchio and Zacur 2012). Recent years have seen an increase in lethal *C. difficile* infections and the emergence of strains with increased toxin production and antibiotic resistance (McDonald et al. 2005; Warny et al. 2005; Razavi et al. 2007; Lessa et al. 2012).

The failure of antibiotics to fully eliminate *C. difficile* leads to recurrent disease episodes in ∼30% of patients (Johnson 2009). This fueled the investigation of fecal microbiota transplantation (FMT) as an alternative treatment option, whereby patients are instilled with healthy donor feces to replenish intestinal microbiota that prevent the growth of *C. difficile*. FMT has shown impressive success rates of ∼90% against RCDIs and no severe adverse effects (Gough et al. 2011; Cammarota et al. 2014; O'Horo et al. 2014). A recent controlled clinical trial demonstrated the superiority of FMT to antibiotics for RCDI treatment (van Nood et al. 2013). FMT led to increased donor-like intestinal bacterial diversities within 2 wk (van Nood et al. 2013). Knowledge about the long-term effects of FMT, however, is presently not available. In addition, previous studies mainly focused on bacteria. Because viruses, especially phages, are the most abundant intestinal entities with the ability to influence microbial communities (Barr et al. 2013; Virgin 2014), they may well be relevant to *C. difficile* infection and the microbial changes following FMT. This is also suggested by recent findings that phages play a causative role in inflammatory bowel disease (IBD), which, similar to RCDI, is characterized by pathologically altered gut microbiota (Norman et al. 2015).

We recently reported on a recovered RCDI patient whose fecal bacteria were of chimeric composition of the patient and the healthy sister donor up to 7 mo post-FMT (Broecker et al. 2013), suggesting that stable attainment of intestinal microbial communities may take longer time periods. Here, we followed up this patient until 4.5 yr post-FMT, characterized fecal bacterial communities by 16S rRNA gene sequencing at various time points, and further analyzed previously reported viromes (Broecker et al. 2013) in comparison to the donor.

## RESULTS

### Patient History

Details on the patient history have been published (Broecker et al. 2013). Briefly, the female patient was 51 years old when admitted to the University Hospital of Zurich with her sixth episode of RCDI, suffering from severe diarrhea and weight loss. Stool samples tested positive for *C. difficile* by standard toxin A and B immunoassays, selective agar cultures, and agglutination assays. The first episode of *C. difficile* infection occurred 2 yr before hospital admission after 7 mo of multiple antibiotic treatment against a complicated jawbone infection.

Multiple rounds of treatments against RCDI with the recommended antibiotics metronidazole and vancomycin, partly in conjunction with the probiotic *Saccharomyces cerevisiae*, were only temporally successful but lead to recurrence after cessation. A therapeutic trial with intravenous immunoglobulins did not induce significant clinical responses. Finally, FMT was performed with donor feces from the patient's sister that tested negative for a variety of bacterial and viral pathogens. A suspension of donor feces in sterile 0.9% sodium chloride solution was applied intra-anally to the patient. Before the treatment, the patient was given vancomycin to suppress growth of pathogenic *C. difficile* as well as loperamide to prevent diarrhea. Following FMT, the patient reported changes in bowel movements and intermittent obstipation, both of which ceased within 10 wk. Ever since, the patient has remained free of symptoms for almost 5 yr now.

### Bacterial Communities of the Patient Were Highly Variable up to 7 mo Post-FMT and Similar to the Donor after 4.5 yr at the Phylum Level

**Figure 1.**
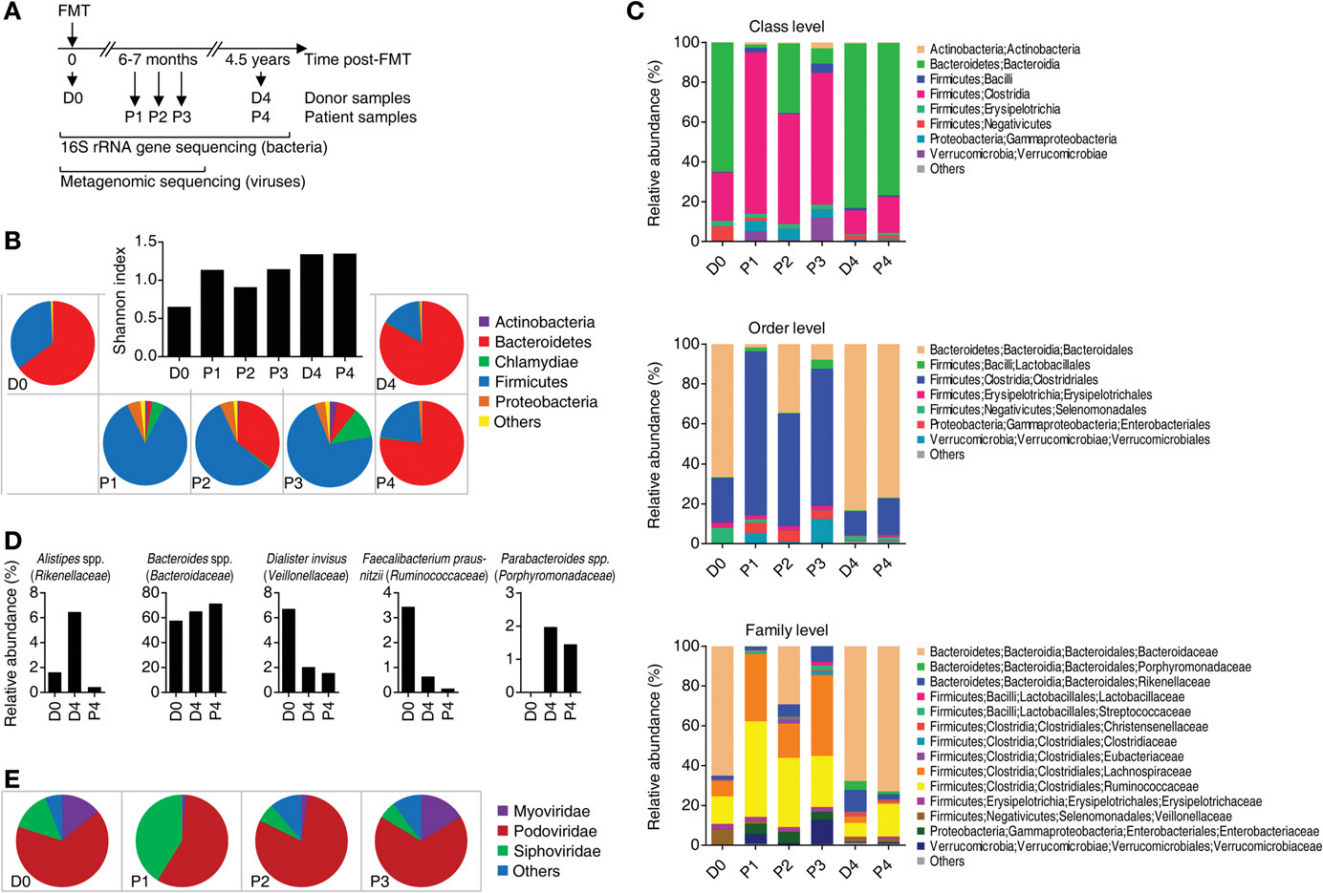
Analysis of fecal microbiota. (*A*) Sample description. Fecal samples of the donor and the patient were collected at the indicated time points and subjected to metagenomic and/or 16S rRNA gene sequencing. FMT, fecal microbiota therapy. (*B*) Bacterial compositions at the phylum level are shown as pie charts. The inlay graph shows bacterial diversities inferred by Shannon indices as bars. (*C*) Bacterial compositions at the class, order, and family levels (from *top* to *bottom*) are shown as stacked bar graphs. Only taxa supported by at least 1% of total reads at each level are shown. (*D*) Relative abundances of the five most dominant bacterial genera in samples D0, D4, and P4 are shown as bar graphs. *Dialister* and *Faecalibacterium* genera were solely represented by the indicated species in each sample. The respective family names are given in parentheses. (*E*) Viral compositions of Caudovirales families are shown as pie charts.

A total of six fecal samples of the patient after FMT and the stool donor were collected at various time points and subjected to 16S rRNA gene sequencing (Fig. 1A). The specimens included two long-term samples of the donor and the patient (D4 and P4, respectively), both collected 4.5 yr post-FMT. Four additional samples, one donor sample at the time of FMT (D0) and three patient samples 6–7 mo after the treatment (P1–P3), have been previously analyzed by metagenomic sequencing (Broecker et al. 2013). In the present study, targeted 16S rRNA gene sequencing was also applied to the latter samples to make them comparable to samples D4 and P4. A summary of the sequencing yields and accuracies is provided in Table 1.

**Table 1.** Summary of 16S rRNA gene-sequencing data

Sample	Date of sampling	Polymerase reads	Polymerase read bases	Polymerase read mean length	Polymerase read quality (%)	Subreads	Subread mean length	Subread N50	Reads of Insert	Reads of Insert mean quality (%)	Mean number of passes	Reads of Insert mean length
D0	Apr 2010	61,513	1,060,087,040	17,233	84.2	742,956	1385	1391	33,448	99.1	17.1	1382
D4	Oct 2014	54,085	824,162,488	15,238	84.2	567,136	1411	1410	28,777	99.1	15.4	1383
P1	Nov 1, 2010	70,286	1,241,608,906	17,665	82.9	842,749	1432	1384	32,708	98.8	16.3	1374
P2	Nov 12, 2010	75,817	1,253,948,163	16,539	83.7	866,330	1405	1404	40,813	99.1	15.6	1373
P3	Nov 25, 2010	95,559	1,693,054,916	17,717	83.6	1,108,225	1485	1414	45,181	99.1	15.6	1373
P4	Oct 2014	70,821	1,211,747,960	17,110	83.3	835,552	1408	1400	38,562	99.1	16.2	1374

Taxonomic analysis of the donor samples at the time of FMT (D0) and 4.5 yr later (D4) revealed bacterial communities dominated by the Bacteroidetes phylum (D0: 65% and D4: 83%), followed by Firmicutes (34% and 15%) (Fig. 1B). Remaining bacteria accounted for <2% in these two samples. In contrast, the patient samples P1–P3 collected 6–7 mo post-FMT were mainly composed of up to 85% (P1) of phylum Firmicutes. In this timespan, bacterial communities underwent extensive fluctuations. For instance, Bacteroidetes comprised 2% (P1), then 35% (P2), and finally 8% (P3) of all bacteria. The phylum Chlamydiae, barely detectable in the donor, constituted up to 12% (P3) of the patient's bacteria. In contrast to P1–P3, the patient sample 4.5 yr post-FMT (P4) mainly contained Bacteroidetes (77%) followed by Firmicutes (21%) and <2% remaining bacteria.

Bacterial diversities were estimated by calculating the Shannon diversity indices for all samples at the species level. The Shannon indices showed a high degree of variability even in the healthy donor, where an about twofold increase from the time point of FMT (D0) to 4.5 yr afterward (D4) was observed (Fig. 1B). In the patient samples, a trend toward increasing diversity from the time period covered by samples P1–P3 to 4.5 yr post-FMT (P4) was observed that may, however, have been due to the fact that samples P1–P3 could not be fully resolved down to the species level. At this time point, the bacterial diversities in the samples from patient (P4) and donor (D4) were highly similar.

### Bacterial Communities of the Patient Were Similar to Those of the Donor after 4.5 yr up to the Order Level but Showed Differences at Lower Taxonomic Levels

More detailed insights into the bacterial communities were gained at lower taxonomic levels. In all six samples, bacteria of the Bacteroidetes phylum were exclusively assigned to the order Bacteroidales (Bacteroidia class) (Fig. 1C). The Firmicutes phylum stratified into the four orders Lactobacillales (Bacilli class), Clostridiales (Clostridia class), Erysipelotrichales (Erysipelotrichia class), and Selenomonadales (Negativicutes class). At the order level, bacterial communities of D4 and P4 remained highly similar.

At the family level, some differences between these two samples became apparent. For instance, the families Porphyromonadaceae and Rikenellaceae, both of the Bacteroidales order as well as the Lachnospiraceae family of the Clostridiales order, were more abundant in D4 than in P4.

Only samples D0, D4, and P4 were compared in more detail at the genus level, because samples P1–P3 provided only full taxonomic information up to the family level. The relative abundances of the five most dominant genera in samples D0, D4, and P4, including two that were only represented by a single species, are shown in Figure 1D. Fractions of *Bacteroides* spp., *Dialister invisus*, and *Parabacteroides* spp. were roughly similar between samples D4 and P4, whereas *Alistipes* spp. and *Faecalibacterium prausnitzii* were more abundant in D4. Most interestingly, we did not identify any sequences assigned to *C. difficile* species in any of the analyzed samples (data not shown).

### Communities of dsDNA Viruses Were Variable and Consisted Mainly of Caudovirales Phages

The analysis of viral dsDNA sequences reported earlier (Broecker et al. 2013) revealed the presence of 22 viruses throughout samples D0, P1, P2, and P3 (Table 2). In each sample, eight to 11 different viruses were identified, mainly belonging to the Caudovirales order (tailed dsDNA phages) that contains the viral families Myo-, Podo-, and Siphoviridae. Most viruses, 14 of 22, were identified uniquely in either sample. Three phages, the *Erwinia* phage vB_EamP-L1 (Podoviridae) and the two Bacteroides phages B124-14 and B40-8 (Siphoviridae), were consistently detected in all four samples and each contained phages of all three Caudovirales groups. Of these, Podoviridae were consistently most abundant. Myo- and Siphoviridae showed highly variable abundances (Fig. 1E). The patient sample P3 contained sequences related to the *Paramecium bursaria Chlorella* virus 1 (PBCV-1) that infects eukaryotic algae (Table 2; Van Etten 2003).

**Table 2.** List of identified intestinal viruses

	Virus	NCBI nucleotide accession number	Genome size (bp)	Number of annotated ORFs	Present in D0?	Present in P1?	Present in P2?	Present in P3?
Myoviridae	*Enterobacteria* phage RB16	NC_014467.1	176,788	271	Yes			Yes
	*Enterobacteria* phage RB43	NC_007023.1	180,500	292	Yes			Yes
	*Klebsiella* phage KP15	NC_014036.1	174,436	258				Yes
	*Bacillus* phage BCP78	NC_018860.1	156,176	227	Yes			
	*Bacillus* phage SP10	NC_019487.1	143,986	236			Yes	
	*Streptococcus* phage EJ-1	NC_005294.1	42,935	73		Yes		
Podoviridae	*Erwinia* phage vB_EamP-L1	NC_019510.1	39,282	51	Yes	Yes	Yes	Yes
	*Escherichia* phage TL-2011b	NC_019445	44,784	57		Yes	Yes	Yes
	*Bacillus* phage ϕ29	NC_011048	19,282	27			Yes	
Siphoviridae	*Bacteroides* phage B124-14	NC_016770	47,159	68	Yes	Yes	Yes	Yes
	*Bacteroides* phage B40-8	NC_011222	44,929	46	Yes	Yes	Yes	Yes
	*Clostridium* phage ϕCP34O	NC_019508	38,309	52		yes		
	*Lactococcus* phage 936 sensu lato	KC182544	27,302	49		Yes		
	*Lactococcus* phage ϕ41	n.a.	n.a.	n.a.		Yes		
	*Listeria* phage 2389	n.a.	n.a.	n.a.	Yes			
	*Listeria* phage B025	NC_009812.1	42,653	65	Yes			
	*Rhodococcus* phage ReqiPepy6	NC_023735	76,797	107	Yes			
Unclassified Caudovirales	*Sinorhizobium* phage PBC5	NC_003324	57,416	83	Yes		Yes	Yes
Unclassified phages	*Clostridium* phage D-1873	n.a.	n.a.	n.a.			Yes	Yes
	*Tetrasphaera* phage TJE1	NC_019930	49,219	66				Yes
Unclassified dsDNA viruses	*Paramecium bursaria* Chlorella virus 1	NC_000852.5	330,611	802				Yes
	*Clostridium* phage ϕSM101	NC_008265.1	38,092	53	Yes			

NCBI, National Center for Biotechnology Information; ORFs, open reading frames; n.a., not available.

## DISCUSSION

FMT has been shown to be a promising treatment option for RCDI patients that leads to replenishment of the patients’ gut microbiota through application of donor feces (Gough et al. 2011; van Nood et al. 2013; Cammarota et al. 2014; O'Horo et al. 2014). Here, we investigated the long-term effects of FMT by analyzing fecal microbiota of a cured RCDI patient in comparison to the donor until 4.5 yr after the procedure.

To analyze the bacterial compositions, we chose 16S rRNA gene sequencing using SMRT (single-molecule real-time) sequencing on the Pacific Biosciences (PacBio) platform. Despite its known limitations regarding raw read quality, this sequencing method has been successfully used previously to resolve compositions of microbial communities (Marshall et al. 2012; Fichot and Norman 2013). In the present study, we used the latest chemistry (C6) that reinforces the main characteristics that make the PacBio platform attractive for microbial sequencing: the absence of sequence context-dependent error and GC-coverage biases (Carneiro et al. 2012; Quail et al. 2012) and, most importantly, the read length. As shown in Table 1, the generated reads were long enough to accurately cover full-length 16S rDNA fragments through the application of the Reads of Insert protocol, an update of the old circular consensus sequence (CCS). The resulting reads were first used as a query against the Silva ribosomal RNA gene database (Quast et al. 2013) using the BLAST algorithm (Altschul et al. 1990). Then, the MEGAN program (Huson et al. 2007) was used to build taxonomic landscapes of the bacterial communities. This workflow has been successfully used before to study microbial communities (Thomas et al. 2012; Li et al. 2013; Sharpton 2014) even of high diversity (Valverde and Mellado 2013). In the present study, both the high-quality scores of the Reads of Insert of >99% and the mean number of passes of at least 15.4 indicated reliable sequencing results (Table 1).

The bacterial composition of the donor was relatively stable and comparable at the time of FMT and 4.5 yr later (Fig. 1B), which is in accordance with the known temporal stability of adult intestinal microbiota (Zoetendal et al. 1998). At the phylum level, Bacteroidetes were most prominent, followed by Firmicutes, typical of healthy gut microbiota (Eckburg et al. 2005). The patient's fecal microbiota underwent extensive compositional fluctuations and were dominated by Firmicutes up to 7 mo post-FMT, suggesting ongoing adaptation processes of donor microbiota in the patient's intestine that may also reflect changes in nutrition over the observation period. This is in accordance with our and other groups’ recent findings that showed high degrees of bacterial variation in RCDI patients up to 7 mo post-FMT (Broecker et al. 2013; Weingarden et al. 2015). However, 4.5 yr post-FMT, the patient's bacteria have attained a donor-like composition at the phylum level, indicating full and stable engraftment of the donor's microbiota. The similarities between the donor's and patient's bacterial compositions remained up to the order level (Fig. 1C). Differences observed at lower taxonomic levels might reflect host-dependent adaptation processes or temporal fluctuations. Of note, four of the five most prominent genera identified in both donor samples as well as the patient sample after 4.5 yr, *Alistipes*, *Bacteroides*, *Dialister*, and *Faecalibacterium* (Fig. 1D), are known constituents of healthy fecal microbiota (Claesson et al. 2011; Joossens et al. 2011). This further indicated that FMT led to healthy and sustainable microbiota in the patient. *Parabacteroides*, another genus typical of healthy fecal microbiota (Claesson et al. 2011) was identified in both long-term samples but not in the donor at the time of FMT, perhaps reflecting temporal fluctuations in the healthy donor. One notable species detected in these three samples is *Faecalibacterium prausnitzii* (Fig. 1D). This species was also detected in the patient samples 6–7 mo post-FMT with abundances of <0.1% (data not shown). *Faecalibacterium prausnitzii* is recognized as one of the most important species of healthy individuals and normally constitutes >5% of the gut microbiota (Miquel et al. 2013). Lower than usual levels of *F. prausnitzii* have been associated with Crohn's disease (Sokol et al. 2008; Joossens et al. 2011). The low abundance of *F. prausnitzii* in the samples of the present study, especially of the patient, is intriguing. However, this may still reflect normal fluctuations, as there have not been any symptoms of Crohn's disease or other apparent complications in the patient.

The fact that the patient's clinical symptoms, which included severe diarrhea in the absence of antibiotic treatment against *C. difficile* (Broecker et al. 2013), resolved promptly after FMT suggests that gut microbiota were able to exert normal metabolic functions even before full engraftment. This may be explained by the fact that the patient's bacterial diversity even during the highly variable time period up to 7 mo post-FMT was already in the range of the healthy donor. In agreement with the absence of symptoms until today, *C. difficile* bacteria were undetectable in the samples of the patient, similar to the donor who tested negative for *C. difficile* before FMT (Broecker et al. 2013). This showed sustainable elimination of *C. difficile* bacteria from the patient's intestine and successful therapy on the molecular level. It is worth mentioning that the patient had to undergo two short-term antibiotic treatments for other indications with apparently no further consequences on the gut microbiota. The finding that the patient's fecal microbiota attained a highly donor-like composition after 4.5 yr suggests that long-term follow-up should be implemented into clinical practice. Moreover, this finding highlights the importance of selecting donor feces with a healthy microbiota composition. An even better source material for FMT could be prospectively freeze-stored own feces. The suitability of frozen feces for FMT has been demonstrated by a recent phase 1 clinical study, in which orally administered frozen capsules containing healthy fecal matter cured 90% of RCDI patients (Youngster et al. 2014).

Viral sequences were identified by metagenomic sequencing from the same DNA preparations that were used for 16S sequencing. The analysis of viral dsDNA sequences from a previous study (Broecker et al. 2013) revealed the presence of Caudovirales phages in all investigated samples of the donor and the patient (Table 2). Caudovirales have been shown before to be the dominant viruses in the human intestine, followed by ssDNA phages of the Microviridae family that we were unable to detect with the metagenomic sequencing approach (Lepage et al. 2008; Norman et al. 2015). Three phages were identified in all of the analyzed samples of the donor and the patient. One is the Podovirus *Erwinia* phage vB_EamP-L1 that infects *Erwinia amylovora* bacteria, the causal agent of fire blight in Rosaceae species including apple and pear trees (Born et al. 2014). As its host, *E. amylovora*, is not a normal constituent of intestinal microbiota, this phage was likely a food contaminant that survived the stomach passage. The other two universally detected phages were the Siphoviruses *Bacteroides* phages B124-14 and B40-8 that infect bacteria of the *Bacteroides* genus abundantly found in the gut of healthy humans (Ogilvie et al. 2012). We detected low and variable quantities of the *Bacteroides* genus in the patient 6–7 mo post-FMT. In percent of total reads, 0.3% (P1), 20.7% (P2), and 0.3% (P3) were assigned to *Bacteroides* (data not shown). Even though 16S rRNA gene sequencing did not fully resolve the genus level in these samples, they matched those of the Bacteroidaceae family that contained only *Bacteroides* spp. in samples D0, D4, and P4 (Fig. 1C). In contrast to P1–P3, both donor samples showed high abundances (57.7% and 61.2%, D0 and D4, respectively) of *Bacteroides* spp. similar to the patient sample 4.5 yr post-FMT (71.4%, P4) (Fig. 1D). The *Bacteroides* phages may have been transferred from the donor to the patient where they could have played a role in controlling *Bacteroides* populations during early stages through bacterial lysis, as suggested before (Lepage et al. 2008; Ogilvie et al. 2012; Norman et al. 2015).

The replication of phages is dependent on the presence of their bacterial host. Therefore, it is not surprising that we found extensive fluctuations in the virome when bacteria were also highly variable in the patient's samples up to 7 mo post-FMT. It has to be noted that the viral abundances presented in Figure 1E were produced by the metagenomic sequencing approach in which viruses with higher gene numbers could be overrepresented (Broecker et al. 2013). The Myoviridae may appear more abundant because of their larger average genomes and gene numbers (Table 2). The relatively small Podoviridae were therefore the most dominant Caudovirales phages among all samples. Phages are known to be able to regulate gut microbiota by bacterial lysis, horizontal gene transfer, and modulation of the intestinal immune system (Barr et al. 2013; Virgin 2014). It is thus tempting to speculate that they may have contributed to the bacterial population dynamics in the patient when the gut microbiota have not yet been fully established.

The identification of PBCV-1-related sequences in one of the patient's samples is intriguing because this virus has not yet been identified in the human intestine. PBCV-1 is a giant virus that contains about 800 open reading frames (ORFs), 400 protein-coding genes, and up to 16 tRNA genes. The related *Acanthocystis turfacea Chlorella* virus 1 (ATCV-1) has been identified recently in human nasopharyngeal samples where its presence correlated with reduced cognitive function (Yolken et al. 2014), showing that giant viruses may well be relevant for human health and disease. Sequences of Phycodnaviridae, the viral family harboring PBCV-1 and ATCV-1, have previously been reported to be present in the intestine of rodents (Phan et al. 2011). A possible role of PBCV-1 in the human intestine remains to be elucidated, but its presence may simply have resulted from the intake of *Chlorella* algae–contaminated freshwater (Van Etten 2003). The first identification of PBCV-1-related sequences in a human intestine, however, suggests that other unexpected viruses may be detected in future studies.

Phages are ∼10-fold more abundant than their prokaryotic hosts in the human gut where they may influence bacterial diversity and population structure (Lepage et al. 2008; Minot et al. 2013). The low number of eight to 11 phages identified per sample here may be an underrepresentation, perhaps attributable to the DNA isolation procedure (Broecker et al. 2013). Also, the sequencing approach did not allow for distinguishing integrated prophages from genomes of free virus particles (Broecker et al. 2013). The isolation of viral genetic material from viral particles in stool supernatants may be more suitable to characterize fecal virus communities (Lepage et al. 2008; Phan et al. 2011; Norman et al. 2015). It has been reported that phages in the gut may be liberated under pathologic conditions, like inflammation (Lepage et al. 2008), suggesting that low numbers may correlate with healthy gut microbiota.

Overall, our findings demonstrate the long-term efficacy of FMT for the treatment of RCDI on the molecular level. Highly diverse phage communities suggest a possible role of phages during engraftment of donor microbiota. In light of a recent study that showed a causative role of phages in the etiology of IBD (Norman et al. 2015), gut viruses may be relevant during *C. difficile* disease and response to FMT as well. This is the subject of ongoing comprehensive investigations of fecal viromes of RCDI and IBD patients treated with FMT at the University Hospital and the ETH Zurich.

## METHODS

### Metagenomic Sequencing

The metagenomic-sequencing data used to characterize viruses in samples D0, P1, P2, and P3 (Table 1) is from a previous publication (Broecker et al. 2013). Briefly, DNA was isolated from ∼0.2 g of frozen-stool samples with the QIAamp DNA Stool Mini Kit (QIAGEN), then treated with DNase-free RNase (Fermentas), and further purified by phenol/chloroform extraction. Barcoded libraries were generated with the NEBNext DNA sample prep kit (New England Biolabs) and sequenced on an Illumina Genome Analyzer IIx instrument in a 120-base paired-end multiplex run. Read sets were assembled to contiguous sequences with the CLC Genomic Workbench V5. ORFs of these contigs were predicted with GLIMMER3 (Salzberg et al. 1998). The ORFs were queried in the NR-PROT protein database (Benson et al. 2009) using BLASTP (Altschul et al. 1990). The first listed protein hit of each ORF was taxonomically assigned using MEGAN. For more details, refer to Broecker et al. (2013).

### 16S rRNA Gene Sequencing

Fecal DNA was isolated as described above in “Metagenomic Sequencing.” For samples D0, P1, P2, and P3, the same DNA preparations used previously for metagenomic sequencing (Broecker et al. 2013) and stored at −80°C were subjected to 16S rRNA gene sequencing. A broad-range 16S rDNA PCR was performed with the Phusion High-Fidelity PCR Master Mix (Finnzymes/NEB), using universal primers TPU-1 (AGAGTTTGATCMTGGCTCAG), 1387r (GGGCGGWGTGTACAAGGC), and one-tenth of primer Bif-8F (AGGGTTCGATTCTGGCTCAG) in order to amplify ∼1300 bp of the bacterial 16S rRNA genes. PCR cycling conditions were 3 min at 98°C (10 sec at 98°C 30 sec at 55°C, 45 sec at 72°C) × 24; 10 min at 72°C). Sequencing on the PacBio RS II was performed with SMRT cell libraries prepared with the DNA Template Prep Kit 2.0 (250 bp to <3 kb) (Pacific Biosciences p/n 001-540-726) and 1400-bp selected target size. Consensus sequences were generated with the Reads of Insert protocol by retaining only sequences with ≥90% accuracy and 1400 ± 100 bp length. The minimum number of passes was set to 3. A summary of the sequencing yields and accuracies is provided in Table 1.

### Phylogenetic Analysis of 16S rRNA Gene Sequencing Data

Resulting Reads of Insert consensus sequences were analyzed in the release 115 of the SILVA database (Quast et al. 2013) using v2.2.29 of BLAST (Altschul et al. 1990). An e-value of 0.001 was imposed as threshold and 100 sequences were retained (‘-evalue 0.001 -max_target_seqs 100’). Output from the BLAST alignment was analyzed with v5.0 of the MEGAN program (Huson et al. 2007) with a threshold of at least five supporting reads for a taxonomic level to be reported as present. Bacterial diversities at the species level have been estimated by calculating Shannon indices (Etter 1999).

## ADDITIONAL INFORMATION

### Ethics Statement

Informed consent has been obtained from the study participants. This study was approved by the Institutional Review Board of the University Hospital Zurich.

### Database Deposition and Access

The sequencing data have been deposited in the NCBI SRA database (http://www.ncbi.nlm.nih.gov/sra) under BioProject ID PRJNA292639 and SRA ID SRP062303. Accession numbers of individual samples are SRX1143098 (D0), SRX1142599 (P1), SRX1143096 (P2), SRX1143095 (P3), SRX1143099 (D4), and SRX1143097 (P4).

### Acknowledgments

The authors thank the donor and patient for their cooperation and their strong support of this study. We are also grateful to Prof. Peter H. Seeberger, Max Planck Institute of Colloids and Interfaces, Potsdam, Germany, for generous support of F.B. We thank colleagues from the Max Planck Institute for Molecular Genetics, Berlin, Germany for their support of this work and access to laboratory infrastructures and in particular to the bioinformatics facilities. The excellent technical assistance of Monique Herensperger is gratefully acknowledged.

### Author Contributions

F.B., J.K., and M.S. performed the laboratory experiments and coordinated the work with G.Ru. who generated the data at the Functional Genomics Centre, Zurich, Switzerland. M.H. was involved in guidance of the patient and the donor, G.Ro. and L.B. are the medical doctors responsible for the clinical and ethical aspects involved. K.M. initiated and coordinated the project. F.B., J.K., and K.M. wrote the manuscript with assistance from G.Ru.

### Funding

We thank colleagues of the Institute of Medical Microbiology, University of Zurich, Switzerland and of the Institute of Food, Nutrition and Health, ETH Zurich, Zurich, Switzerland for their generous financial support. K.M. supplied some private funds.

### Competing Interest Statement

The authors have declared no competing interest.
